# Role of miR-182/PDCD4 axis in aggressive behavior of prostate cancer in the African Americans

**DOI:** 10.1186/s12885-021-08723-6

**Published:** 2021-09-15

**Authors:** Marisa Shiina, Yutaka Hashimoto, Priyanka Kulkarni, Pritha Dasgupta, Varahram Shahryari, Soichiro Yamamura, Yuichiro Tanaka, Rajvir Dahiya

**Affiliations:** Department of Urology, Urology Research Center, Veterans Affairs Medical Center and University of California San Francisco School of Medicine (UCSF), 4150 Clement Street, San Francisco, CA 94121 USA

## Abstract

**Background:**

Prostate cancer is one of the most commonly diagnosed cancers among men. African Americans (AA) are at an increased risk of developing prostate cancer compared to European Americans (EA). miRNAs play a critical role in these tumors, leading to tumor progression. In this study, we investigated the role of miR-182 in racial disparity in prostate cancer.

**Results:**

We found significantly increased levels of miR-182 in prostate cancer tissues compared to BPH. Also, miR-182 shows increased expression in AA prostate cancer cell line and tissue samples compared to EA. We performed biochemical recurrence (BCR) - free survival time in AA and EA patients and found that high miR-182 expression had significantly shorter BCR-free survival than patients with low miR-182 expression (*P* = 0.031). To elucidate the role of miR-182, we knocked down miR-182 in EA (DU-145 and LNCaP) and AA (MDA-PCa-2b) cell lines and found an increase in apoptosis, arrest of the cell cycle, and inhibition of colony formation in the AA cell line to a greater extent than EA cell lines.

**Conclusions:**

Our results showed that PDCD4 is a direct miR-182 target and its inhibition is associated with aggressiveness and high Gleason grade in prostate cancer among AA. These findings show that miR-182 is highly expressed in AA patients and miR-182 may be a target for effective therapy in AA patients.

**Supplementary Information:**

The online version contains supplementary material available at 10.1186/s12885-021-08723-6.

## Introduction

Prostate cancer is the second leading cause of cancer death in men with an estimated 33,330 deaths in 2020 [1]. African Americans (AA) have a 74% higher incidence of developing prostate cancer and are more than twice as likely to die of the disease than European American (EA) men [2, 3]. It is known that socioeconomic, educational, cultural, access to health care, and genetic factors contribute to racial disparities in prostate cancer incidence and outcome [4–6].

Although there have been many advances in the treatment of urological cancers, treatment often results in tumor recurrence and therapy failure. Also, a major challenge in prostate cancer treatment is the lack of effective biomarkers. Reliable diagnostic and prognostic biomarkers in cancer are urgently needed [7, 8]. MicroRNAs (miRNA) are small non-coding regulatory RNAs of 18–24 nucleotides in length which bind to complementary sequences of mRNA, regulating post-transcriptional gene expression, and playing a pivotal role in tumorigenesis. miRNAs have been tested as potential biomarkers in cancer research [9–11]. miR-182, together with miR-183 and miR-96, belong to a miRNA cluster located on chromosome 7q31–34 and share similar seed sequences [12]. miR-182 is shown to be a cancer-related oncogenic miRNA that is dysregulated in various cancer tissues, including breast, lung, skin, ovarian, prostate, brain, and colorectal cancers [12–15].

Programmed cell death 4 (PDCD4) was first identified as a gene regulating apoptosis [16]. It functions as a tumor suppressor and is down-regulated in different types of cancers [17]. PDCD4 is lost in certain aggressive human carcinomas of the lung, breast, colon, and prostate [14, 18–20]. Loss of PDCD4 is associated with biological aggressiveness and poor prognosis in different cancer types [21, 22].

The involvement of miRNA expression in racial disparity was first described by Calin *et. al*. [23]. They showed that miR-301, miR-219, miR-26a, miR-1b-1, and miR-30c-1 are at least three times differentially expressed in African Americans compared to Caucasians in normal prostate tissue.

In this study, we investigated the role of miR-182 in racial disparity in prostate cancer. We found higher expression of miR-182 in an AA prostate cancer cell line and tissue samples compared to EA. Also, PDCD4 is a direct miR-182 target and its inhibition is associated with aggressiveness and high Gleason grade in prostate cancer among AA.

## Materials and methods

### Patient samples and cell lines

Human prostate cancer cell lines MDA-PCa-2b (ATCC/CRL-2422) (doubling time 45 h), the only available AA cell line at American Type Culture Collection (ATCC), DU-145 (ATCC/HTB-81) (doubling time 30 h), and LNCaP (ATCC/CRL-1740) (doubling time 60 h) were obtained from the ATCC. The EA-derived cell lines, DU-145 and LNCaP, were maintained in RPMI-1640 with 10% FBS and the AA-derived cell line, MDA-PCa-2b, was cultured in HPC1 (Athena ES) with 10% FBS in Poly-L-Lysine (Sigma–Aldrich) [24] coated culture dishes. The culture medium was supplemented with antibiotics and cells were cultured at 37 °C with 5% CO_2_. Paired tumor and adjacent normal tissue samples from EA (*n* = 42), AA (*n* = 40) clinical FFPE (Formalin Fixed Paraffin Embedded), and benign prostatic hyperplasia (BPH) (*n* = 24) were obtained from the Veterans Affair Health Care System, San Francisco, CA, USA. Written informed consent was obtained from all patients, and the study was in accordance with institutional guidelines. FFPE block sections were H&E stained and reviewed by a board-certified pathologist at the SFVA Medical Center to mark the normal and cancer regions (roadmaps). Based on the identification of normal and cancer regions, the slides were microdissected for nucleic acid extractions. Nanodrop (Thermo Fisher Scientific) was used for RNA concentration and quality check [25]. miRNA expression data from The Cancer Genome Atlas Prostate Adenocarcinoma (TCGA PRAD) was also used.

### Quantitative real-time reverse transcription-polymerase chain reaction

Total RNA was isolated using a miRNeasy mini kit (Qiagen) and reverse-transcribed into cDNA with the SuperScript III kit (Life Technologies) as described previously [26]. For miR-182 expression analysis, cDNA was synthesized from total RNA with the TaqMan Reverse Transcription kit (Applied Biosystems) with specific primers and the cDNA was subjected to TaqMan Probe-based Real-Time PCR using TaqMan miRNA assays Universal PCR Master Mix (Thermo Fisher Scientific) according to the manufacturer’s instructions. Real-time reverse transcription-polymerase chain reaction (RT–PCR) was performed with SYBR Green (Applied Biosystems) using a QuantStudio 7 PCR System. The expression levels of miRNA were calculated as the amount of target miRNA relative to that of RNU48 control to normalize the initial input of total RNA.

### Generation of stable cells and confirmation of anti-miR-182 expression level

The Lentivirus miRNA Inhibitors system was used to carry out stable miR-182 loss-of-function studies. A total of 7.5 μg of psPAX2 and 2.5 μg of pMD2.g plasmids were used to pack 10 μg of constructed pLenti-III-mir-Off viral vector (Lenti-miR-Off-hsa-miR-182-5p and pLenti-III-mir-Off Control vectors (Cat. #mh30241 & m007); ABM). Stable cells were generated as described previously [27]. We used 293 T cells for packaging the anti-miR-182 and control lentiviral vector. Infected vectors were used for infection of cancer cell lines for 3 days. Selection of stable cells was done with 5 μg/mL puromycin for 1–4 weeks. Antisense miR-182 expression was confirmed by qPCR using a custom TaqMan small RNA assay kit (Target sequence; AGUGUGAGUUCUACCAUUGCCAAA, Cat. #4440418, Thermo Fisher Scientific) according to the manufacturer’s protocol.

### PDCD4 transient transfection

To knockdown PDCD4 expression, miR-182 inhibited MDA-PCa-2b cells were transfected with a PDCD4 Human siRNA Oligo Duplex (OriGene, SR309053) using Lipofectamine RNAi Max (Thermo Fisher Scientific) at 20 nM for 48 h.

### Dual-luciferase reporter assay

In the 3′-UTR luciferase reporter assay, the wild-type (WT) luciferase reporter of the PDCD4 gene was generated by annealing the forward and reverse oligonucleotides and then cloned into pmiR-GLO reporter vector (Promega). Oligonucleotides for the PDCD4 3′-UTR were as follows: WT 5′- AAACTGTGCTAATTTAAACTGCCAAATT-3′ (forward) and 5′-CTAGAATTTGGCAGTTTAAATTAGCACAGTTT-3′ (reverse). Anti-miR-182-stable MDA-PCa-2b cells were transfected with a pmiR-GLO vector containing the 3’UTR sequences and luciferase activity was measured with the Promega Dual-Luciferase Reporter kit (Promega) according to the manufacturer’s protocols after transfection. Relative Renilla luciferase activity was normalized to firefly luciferase activity.

### Apoptosis assay

Apoptosis assay was performed as described previously [26]. Stable transfected cells with miR-182 knockdown or negative control were stained with PE Annexin V and 7AAD viability dye (PE Annexin V apoptosis detection kit, Beckman Coulter). After incubation, cells were analyzed using BD FACSVerse (BD Pharmingen).

### Cell cycle and colony formation assays

For the cell cycle, samples were collected and fixed in 70% ethanol overnight. Fixed cells were stained with PI/RNase Staining Buffer (BD Biosciences) and analyzed by FACSVerse (BD Biosciences). Clonogenic assay was performed by seeding 250 cells/well in 24-well tissue culture plates. After incubation for 10–14 days, colonies were stained with crystal violet and quantified by using ImageJ software. Each experiment was performed independently in triplicate.

### Western blot analysis

Cells were lysed with RIPA buffer (Thermo Scientific) containing Halt Protease and Phosphatase Inhibitor Cocktail (Thermo Scientific). Protein concentration was determined using the BSA Protein Assay (Thermo Fisher Scientific). Western blots were performed using NuPAGE 4–12% Bis-Tris Protein Gels (Invitrogen) as described before [26]. Briefly, gels were run in MES buffer (Invitrogen) and wet transferred onto the nitrocellulose transfer membrane. The following primary antibodies were used: PDCD4 (Cell Signaling, 9535), and ß-actin (Cell Signaling, 3700). Goat anti-rabbit IgG (H + L) 800 CW or goat anti-mouse (H + L) 680RD was applied for 45 min at room temperature (1: 25000, LI-COR) before washing with PBS containing Tween 20. Blots were imaged using an Odyssey Infrared Imaging System Scan and quantification was carried out with the LI-COR Odyssey® scanner and software (LI-COR Biosciences).

### Immunohistochemical analysis (IHC)

Tissue sections from paraffin-embedded human prostate cancer specimens were stained with antibodies against PDCD4. IHC analyses were performed using The Vision UltraVision Detection System (Thermo Fisher Scientific). The slides were visualized using 3,3-diaminobenzidine tetrahydrochloride plus (DAB+). Counterstaining for nuclei was performed using hematoxylin. The staining intensity score was calculated using ImageJ software.

### Statistical analysis

**Fig. 1:**
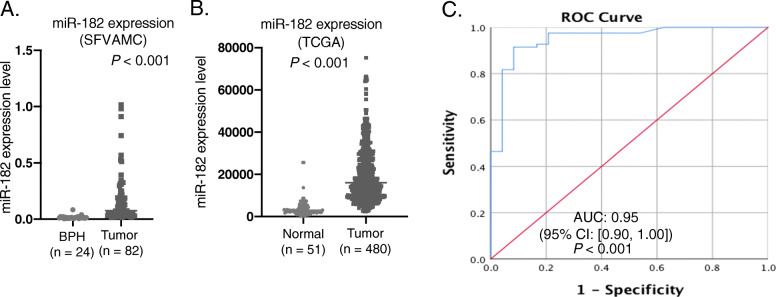
miR-182 expression and ROC curve in BPH and prostate cancer patients. **A** qPCR analysis for miR-182 expression in BPH (*n* = 24) and prostate cancer tissues (*n* = 82) normalized to RNU48 (*P* < 0.001) from the SFVAMC cohort. **B** analysis of miR-182 levels on normal (N) and prostate tumor samples (T) from TCGA data. **C** ROC curve analysis between BPH and tumor samples from the SFVAMC cohort

**Fig. 2:**
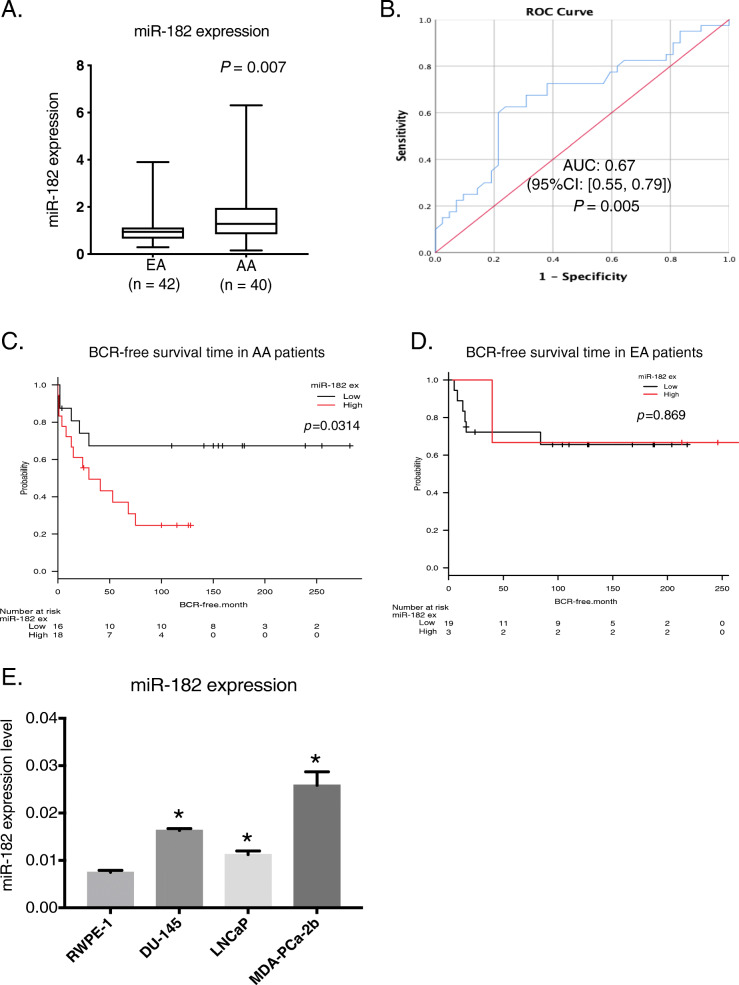
miR-182 expression between AA and EA patients from the SFVAMC cohort. **A** qPCR analysis for miR-182 expression in EA (*n* = 42) and AA (*n* = 40) from the SFVAMC cohort. **B** ROC curve analysis between AA and EA patient samples. **C** and **D** Biochemical recurrence (BCR)-free survival curves for two groups defined by low and high expression of miR-182 in AA (**C**) or EA (**D**) patients with prostate cancer. **E** miR-182 expression levels in prostate cell lines RWPE-1, DU-145, LNCaP, and MDA-PCa-2b assessed by qPCR. *, *P* < 0.05. Data are presented mean ± SD

Analyses were performed using GraphPad Prism 8 and IBM SPSS Statistics, version 26. Two-tailed Student’s *t-test* was used for comparisons between groups. Mann- Whitney test was used to assess the difference between miR-182 expression in BPH or normal/tumor clinical tissues (Fig. 1A and B) and EA/AA samples (Fig. 2A). Kaplan-Meier curve, log-rank test, uni- and multivariate analysis were computed by EZR [28]. Tests with *P* values less than 0.05 were considered statistically significant.

## Results

### Diagnostic potential of miR-182 expression in prostate cancer

To determine the role of miR-182 in prostate cancer, we first determined the miR-182 expression by quantitative real-time RT-PCR in BPH (*n* = 24) and prostate cancer patient samples (*n* = 82) from the SFVAMC cohort. We found significantly increased levels of miR-182 in prostate cancer tissues compared to BPH (*P* < 0.001) (Fig. 1A). Also, we validated the expression profile in a cohort from TCGA PRAD and found that miR-182 expression is significantly higher in tumors compared to normal samples (*P* < 0.001) (Fig. 1B). We then performed a ROC curve analysis of BPH and tumor samples to determine if miR-182 can be used as a diagnostic parameter in prostate cancer. ROC curve from BPH and prostate cancer patient samples from the SFVAMC cohort showed that miR-182 can discriminate between BPH and tumor tissues with an area under the curve (AUC) of 0.95 (95% CI: [0.90, 1.00]; *P* < 0.001) (Fig. 1C). This data suggests that miR-182 possesses significant potential as a molecular biomarker and can discriminate between prostate cancer and benign conditions.

### Higher expression of miR-182 in an African American prostate cancer cell line and tissue samples compared to European Americans

We next analyzed if the miR-182 expression is associated with clinicopathological parameters such as PSA, race, biochemical recurrence, age, T-classification, and Gleason grading [29]. Increased miR-182 expression was significantly upregulated in the high PSA level (*P* = 0.030). Interestingly, we found that AA showed a higher expression of miR-182 compared to EA samples (*P* = 0.017). the miR-182 expression level was not associated with age, T-classification, or Gleason grades (Table 1). This suggests that miR-182 expression could potentially be associated with PSA level and biological racial differences between AA and EA prostate cancer.

**Table 1:** miR-182 expression and clinicopathological characteristics of prostate cancer patients

	tissue (n=82)	miR-182 (mean±SD)	P value
PSA (<10; >10)			
<10	55	1.145±0.774	**0.030**
>10	27	2.090±0.402	
European-american	42	1.087±0.750	**0.017**
African-american	40	1.844±1.806	
Biochemical recurrence			
No	39	1.270±1.380	0.278
Yes	27	1.678±1.636	
Age			
<65	44	1.216±0.911	0.061
≥65	38	1.877±1.947	
T-classification			
T2	47	1.125±0.824	0.096
T3/T4	21	1.507±0.946	
Gleason			
Grade1/Grade2	51	1.138±0.995	0.060
Grade3/Grade4/Grade5	23	2.040±2.114	

Fig. 2A shows expression analysis of miR-182 from 40 AA and 42 EA prostate patient samples by qRT-PCR and this miRNA was significantly higher in AA samples compared to EA samples (*P* = 0.007). Considering the aggressiveness of miR-182 in AA patients, we performed ROC curve analyses based on miR-182 expression in EA and AA tumor samples. Our analyses showed that miR-182 can discriminate between EA and AA tissues with an area under the ROC curve of 0.67 (95%CI: [0.55, 0.79], *P* = 0.005) (Fig. 2B). Further, to evaluate the possible prognostic value of miR-182, we performed BCR-free survival time in AA and EA patients. We dichotomized the samples in the low expression group (≤1.2 fold) and high expression group (> 1.2 fold). Figure 2C shows results for AA patients and we found that high miR-182 expression had significantly shorter BCR-free survival than patients with low miR-182 expression (*P* = 0.031). There was no significant difference in BCR-free survival time in EA patients (Fig. 2D). Furthermore, the univariate analysis with the Cox proportional hazards model found that miR-182 expression was significantly associated with BCR-free survival in AA patients but not in EA patients. PSA levels tended to associate with BCR-free survival time in AA and EA patients (Table 2). This shows that miR-182 is an independent prognostic factor for predicting poor BCR-free survival in AA patients with prostate cancer. It confirms that miR-182 overexpression is a risk factor for poor prognosis in AA patients with prostate cancer.

**Table 2:**
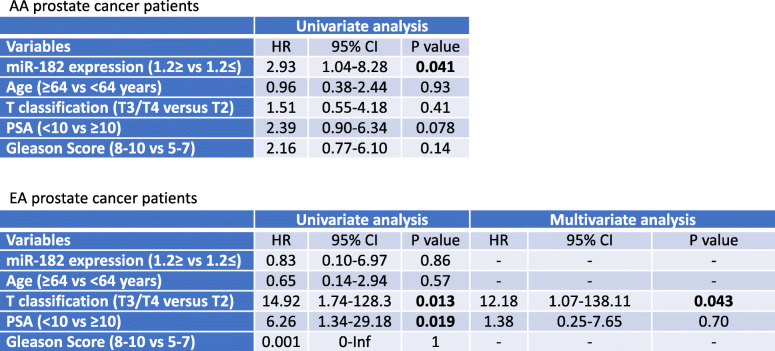
Univariate and multivariate analyses of factors for predicting biochemical recurrence (BCR) in prostate cancer patients (Cox proportional hazards regression model)

To mimic the tissue samples results and help us identify mechanisms related to racial disparity, we analyzed the basal expression level of miR-182 in different cell lines and selected EA cell lines, DU-145 and LNCaP, and the AA cell line, MDA-PCa-2b, which express higher levels of miR-182 than EA cell lines. All cancer cell lines showed significantly higher miR-182 expression compared to *immortalized normal* human prostate cell line, RWPE-1, confirming the oncogenic function of miR-182 (Fig. 2E).

### miR-182 knockdown increases apoptosis, arrests cell cycle, and inhibits colony formation in an African American cell line to a greater extent than European American cell lines

**Fig. 3:**
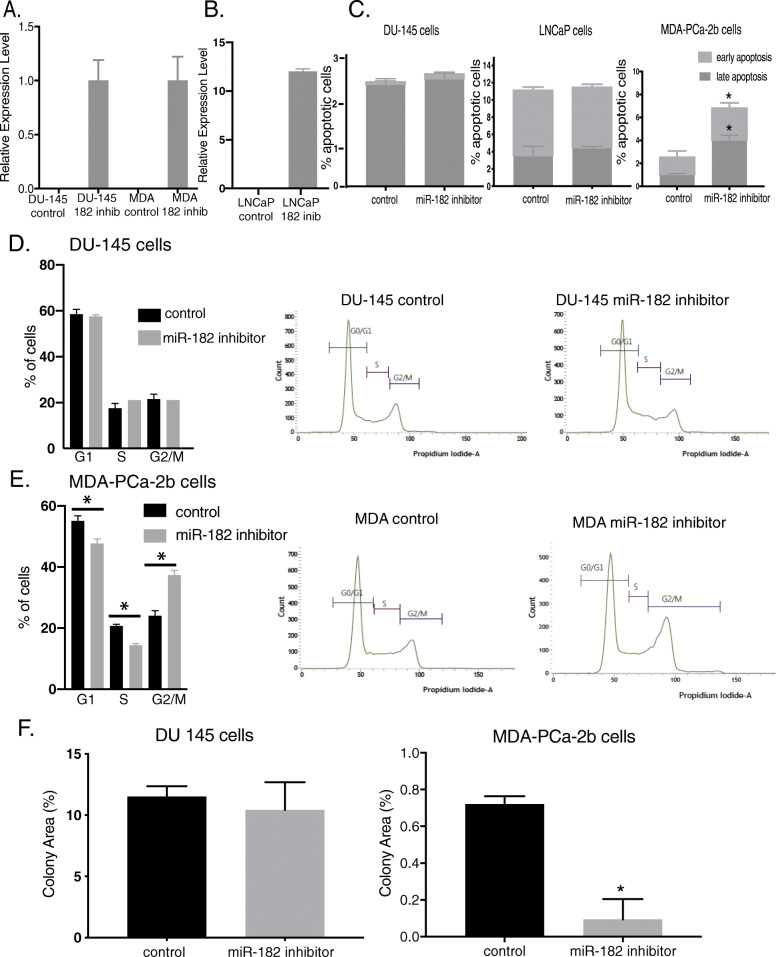
Effect of miR-182 knockdown on apoptosis, cell cycle, and colony formation. **A** and **B** Stable inhibitor of miR-182 in DU-145 or MDA-PCa-2b (**A**) or LNCaP (**B**) knockdown cells by lentiviral vectors were evaluated by qPCR analysis using antisense miRNA. **C** Apoptosis assay in DU-145, LNCaP, and MDA-PCa-2b after the miR-182 knockdown. **D** and **E** Determination of cell cycle distribution after miR-182 knockdown of DU-145 (**D**), and MDA-PCa-2b (**E**), respectively, analyzed by propidium iodide staining by flow cytometry. **F** Clonogenic ability of DU-145 and MDA-PCa-2b after miR-182 knockdown determined by colony formation assay. Bar = ±SD. *, *P* < 0.05

To elucidate the role of miR-182 and to determine whether miR-182 promotes an oncogenic phenotype, we performed a knockdown functional assay in EA (DU-145 and LNCaP) and AA (MDA-PCa-2b) cell lines. We confirmed the knockdown of miR-182 by qRT-PCR using a custom TaqMan miR-182 antisense probe and primers (Fig. 3A and B). Interestingly, knockdown of miR-182 significantly increased apoptosis in MDA-PCa-2b and had no significant effect in DU-145 and LNCaP cells (Fig. 3C). We investigated the effects of miR-182 knockdown on cell cycle progression of DU-145 and MDA-PCa-2b cell lines. miR-182 knockdown in DU-145 cells did not change the cell cycle profile, however, knockdown of miR-182 of MDA-PCa-2b significantly increased the percentage of cells in the G2 phase compared to control cells (Fig. 3D and E). Also, we examined the effects of miR-182 on clonogenicity using colony formation assay. After the miR-182 knockdown, there was no difference in colony formation in DU-145 cells and a significant decrease in colony formation in MDA-PCa-2b cells compared to control miR-transfected cells (Fig. 3F). These data suggest that miR-182 plays a role in apoptosis, cell cycle, and colony formation in AA but not EA cells.

### miR-182 direct targets PDCD4 and is associated with aggressiveness in prostate cancer

**Fig. 4:**
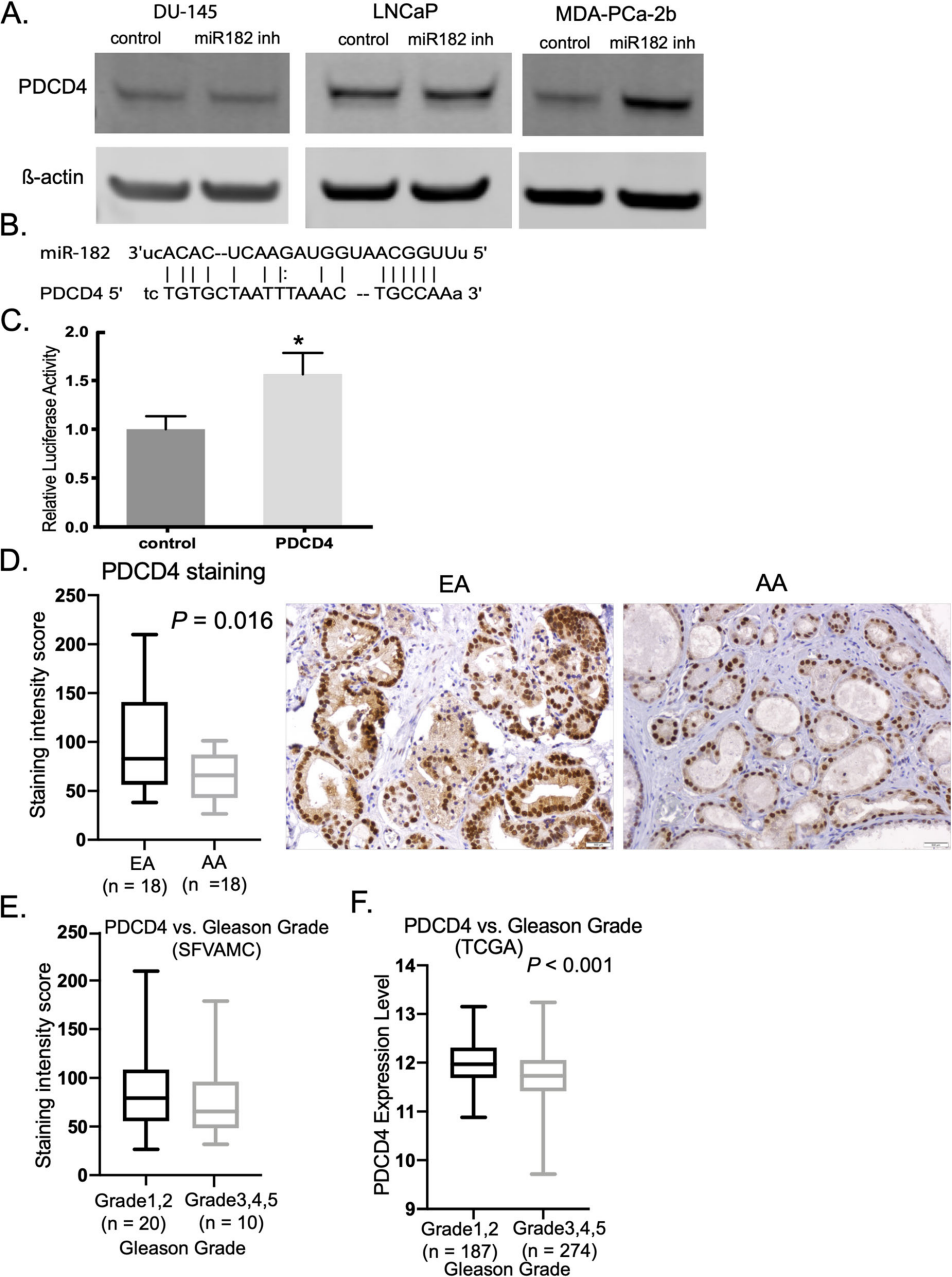
miR-182 targets PDCD4 and contributes to aggressiveness in AA prostate cancer patients. **A** PDCD4 expression levels in prostate cell lines DU-145, LNCaP, and MDA-PCa-2b transfected with miR-182 inhibitor assessed by Western blot. **B** PDCD4-binding site for miR-182. **C** Luciferase reporter assay using vectors containing PDCD4-binding site. **D** PDCD4 staining by IHC in EA and AA patients. **E** and **F** PDCD4 expression in SFVAMC (**E**) or TCGA (**F**) patients with high and low Gleason grade. Grade 1: Gleason score 6 or less; Grade 2: Gleason score 3 + 4; Grade 3: Gleason score 4 + 3; Grade 4: Gleason score 8; Grade 5: Gleason scores 9–10 [29]. *, *P* < 0.05. Data are presented mean ± SD in **C**

Using in silico miRNA target prediction software (TargetScan, miRTarBase, and miRDB), we found several potential targets for miR-182. A large number of potential target sites exist for any given miRNA, among miR-182 targets [30, 31]. We run western for potential targets of miR-182 and found higher expression of PDCD4 after the miR-182 knockdown in MDA-PCa-2b cells. Inhibition of miR-182 in MDA-PCa-2b cells resulted in a significant increase in PDCD4 protein levels, whereas, there were no significant differences in PDCD4 protein levels in DU-145 and LNCaP cells (Fig. 4A). To determine whether miR-182 targets PDCD4, we cloned a PDCD4 3’UTR sequence containing the predicted miR-182 binding site for luciferase reporter assays. miR-182 inhibition caused an increased activity of 3′-UTR luciferase reporter of PDCD4 gene compared to the deletion vector (Fig. 4B and C). Therefore, PDCD4 is a direct target of miR-182. To confirm the levels of PDCD4 protein in AA and EA patients, the expression of PDCD4 was detected in FFPE samples using the IHC technique. EA patients expressed high levels of PDCD4 while weak staining was observed in the AA patient tissues (Fig. 4D). This suggests that the interaction of miR-182 and PDCD4 is associated with prostate cancer aggressiveness in AA patients. Moreover, to determine whether PDCD4 correlates with Gleason grade, whose higher grade indicates increased tumor aggressiveness and the likelihood of disease recurrence, we examined the association of PDCD4 expression and Gleason grade. Although PDCD4 was not significantly correlated with Gleason grade in our VA samples, higher PDCD4 expression trended toward patients with lower Gleason grade, while low PDCD4 expression trended towards higher Gleason grade patients (Fig. 4E). We validated this correlation with TCGA PRAD data and found a significant correlation where high Gleason grade patients show low PDCD4 expression (Fig. 4F). Collectively, our results show that PDCD4 is a direct miR-182 target and is associated with AA aggressiveness and high Gleason grade in prostate cancer.

### Interaction of miR-182 and PDCD4 is essential to inhibit apoptosis

**Fig. 5:**
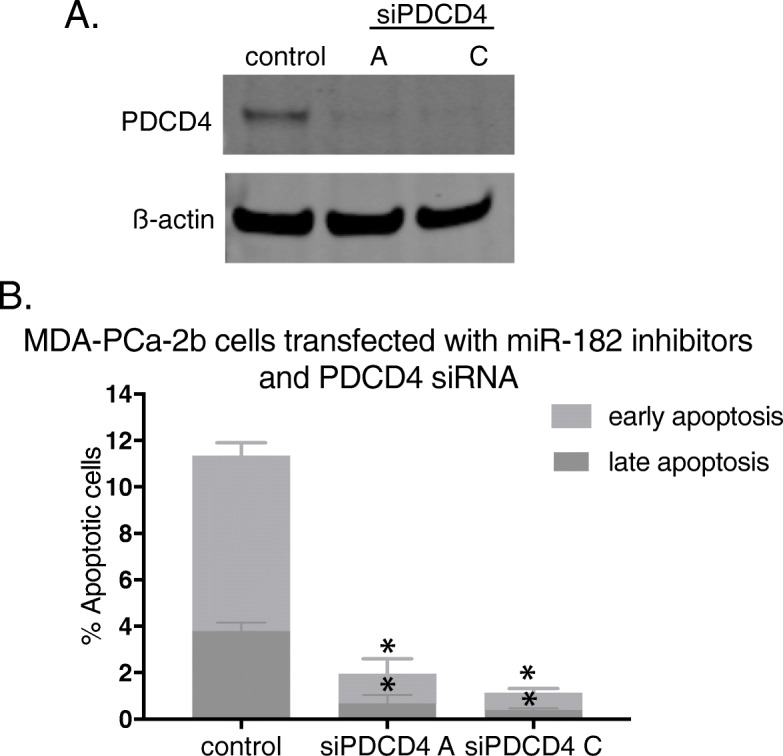
Interaction of miR-182 and PDCD4 is essential for apoptosis. **A** PDCD4 expression in miR-182 knockdown MDA-PCa-2b cells in combination with PDCD4 siRNAs (A and C) analyzed by Western blot. **B** Apoptosis assay for miR-182 knockdown MDA-PCa-2b cells in combination with PDCD4 siRNAs (A and C). *, *P* < 0.05. Data are presented mean ± SD

To evaluate the role of miR-182 and PDCD4 in apoptosis, we knocked down PDCD4 in miR-182 inhibited MDA-PCa-2b cells. Figure 5A shows the PDCD4 expression after PDCD4 siRNA was transfected into miR-182 inhibited MDA-PCa-2b cells. There was a complete knockdown of PDCD4 expression using 2 siRNAs (A and C). Furthermore, apoptosis was significantly inhibited after the knockdown of PDCD4 (Fig. 5B). These results in combination with the results in Fig. 3C show that miR-182 and PDCD4 interact to inhibit apoptosis.

## Discussion

Prostate cancer has a higher incidence in AA compared to EA and is associated with poor prognosis and high mortality in AA [1–3, 32]. It is known that miRNAs are involved in the initiation and progression of various cancers [20, 33, 34]. However, differences in the levels of miRNA expression in tumors of AA in comparison to EA are not well known. We and others have previously shown differences in the expression of miRNAs between AA and EA. [26, 27, 35] We used DU-145, LNCaP, and MDA-PCa-2b, the only available AA cell line at ATCC. We selected these cell lines that express different levels of miR-182 to identify mechanisms related to racial disparity. With the cell line results, we could investigate PDCD4 levels in EA and AA prostate patient samples to determine whether PDCD4 has clinical relevance.

In this study, our results showed significantly increased levels of miR-182 in prostate cancer tissues compared to BPH (Fig. 1A), and analysis of miR-182 expression with clinicopathological parameters showed that miR-182 is correlated with racial disparity (Table 1). AA prostate cancer patients have a higher expression of miR-182 compared to EA patients (Table 1 and Fig. 2A). miR-182 has been reported to be an oncogenic miRNA in different cancers [13, 36–38]. In this study, we showed that miR-182 is highly expressed in AA compared to EA, and knockdown of miR-182 leads to apoptosis, cell cycle arrest, and inhibition of colony formation in AA (Fig. 3). Normal AA prostate cell is not available commercially, so we could not use agomirs to mimic miR-182 expression. Some studies show that G1 arrest plays an important role in colony formation [39, 40]. Our results showed an increase in the G2 phase while decreasing G1 and S-phases. These could explain the decrease in colony formation in MDA-PCA-2b cells.

Xue et al. showed that miR-182 expression is higher in glioblastoma compared with normal human brain tissues and also showed that glioblastoma grade IV cell lines express higher levels of miR-182 compared to grade III-derived glioma cell lines [38]. Yu *et. al.* showed that overexpression of miR-182 promotes breast tumor invasion and TGFβ-induced bone metastasis [41].

The underlying molecular mechanisms of miR-182 regulation in cancer are not clear. Programmed cell death 4 (PDCD4) gene was first reported as homologous to the mouse gene (*MA-3/Pdcd4/A7–1*) which was associated with apoptosis [16, 42, 43]. The regulation of PDCD4 by non-coding RNAs has been less explored. Studies show that overexpression of miR-182 leads to chemoresistance of non-small cell lung cancer to cisplatin by downregulating PDCD4 [44]. miR-183, another member of the miR-182 family, also targets PDCD4 and induces cell proliferation, migration, and invasion in a pancreatic cancer cell line [45]. Our results also show that PDCD4 is a direct target of miR-182 (Fig. 4C) and regulates apoptosis (Figs. 3C and 5B). Moreover, we found that PDCD4 was downregulated in AA compared to EA cell lines and tissues (Fig. 4A and E). Interestingly, we found that low PDCD4 expression is associated with higher Gleason grade (Fig. 4E and F) suggesting that PDCD4 is associated with racial disparity and prostate cancer aggressiveness. Similar observations showed that PDCD4 expression is associated with the histological grade of endometrioid endometrial carcinoma (EEC) where PDCD4 levels in G1 EEC tissues were higher compared with the G2/3 EEC group [22]. Also, it was found that high expression of miR-21 and down-regulation of PDCD4 expression was associated with the aggressive progression and poor prognosis of stage II esophageal carcinoma [46].

Clinical trials for miRNAs have been approved and hundreds of cancer-focused clinical trials involving miRNAs as novel biomarkers or therapies are proceeding towards initiation and completion of potential phase 3 and 4 trials [47–49]. Many miRNAs have been identified as biomarkers for patients at early stages and prediction of prognosis in different cancers [27, 50, 51]. Boubaker *et.al* [46]. showed that miR-182, miR-205, miR-27a, and miR-369 can be used as diagnosis and prognosis biomarkers in patients with urinary bladder cancer. It is also known that miRNAs play a role in cancer drug resistance. miRNA profiling studies in chemoresistant models can be used as predictive biomarkers. Qin et al. described that upregulated miR-182 increases drug resistance in cisplatin-treated hepatocellular carcinoma by regulating TP53INP1. miR-182 may play a role in chemoresistance in prostate cancer and is an interesting area to be investigated. Also, prostate cancer activates the androgen signaling pathway resulting in uncontrolled proliferation of tumor cells [52]. Experiments with miR-182 knockdown in the presence of androgens will be investigated. Although we investigated the role of miRNA and PDCD4 in cell lines and prostate patient tissues, the animal study is a limitation in the present study and the correlation between miR-182 and PDCD4 status should be further investigated in a larger cohort.

In summary, we found higher expression of miR-182 in an AA prostate cancer cell line and tissue samples compared to EA and miR-182 directly targets PDCD4 and is associated with aggressiveness in prostate cancer. Also, we found that miR-182 knockdown increases apoptosis arrests the cell cycle, and inhibits colony formation in an AA cell line more than EA cell lines. Our results suggest that miR-182 may be a clinical biomarker for prostate cancer diagnosis and may be a target for effective therapy in AA patients.

## Supplementary Information

**Additional file 1.**

## Data Availability

The datasets used and/or analyzed during the current study are available from the. the corresponding author on reasonable request.

## Abbreviations

miR-182: MicroRNA-182
PDCD4: Programmed cell death 4
AA: African Americans
EA: European Americans
BPH: Benign prostatic hyperplasia
miRNA: MicroRNA
FFPE: Formalin Fixed Paraffin Embedded
TCGA PRAD: The Cancer Genome Atlas Prostate Adenocarcinoma
cDNA: Complementary Deoxyribonucleic Acid
RT–PCR: Reverse transcription-polymerase chain reaction
WT: Wild-type
PBS: Phosphate-buffered saline
PE: Phycoerythrin
FACS: Fluorescence-activated cell sorting
RIPA: Radioimmunoprecipitation assay
BSA: Bovine serum albumin
IHC: Immunohistochemical analysis
DAB+: 3,3-diaminobenzidine tetrahydrochloride plus
SFVAMC: San Francisco Veterans Affairs Medical Center
PSA: Prostate-specific antigen
ROC: Receiver operating characteristic
siRNA: Small interfering ribonucleic acid
EEC: Endometrioid endometrial carcinoma
