# Supplementary material for: Role of miR-182/PDCD4 axis in aggressive behavior of prostate cancer in the African Americans
Source: BMC Cancer. 2021 Sep 15;21:1028. doi: 10.1186/s12885-021-08723-6 (PMC8444584; doi:10.1186/s12885-021-08723-6)

Supplemental Figure 1: Original Western blot images for A (Figure 4A) and B (Figure 5A)

A.

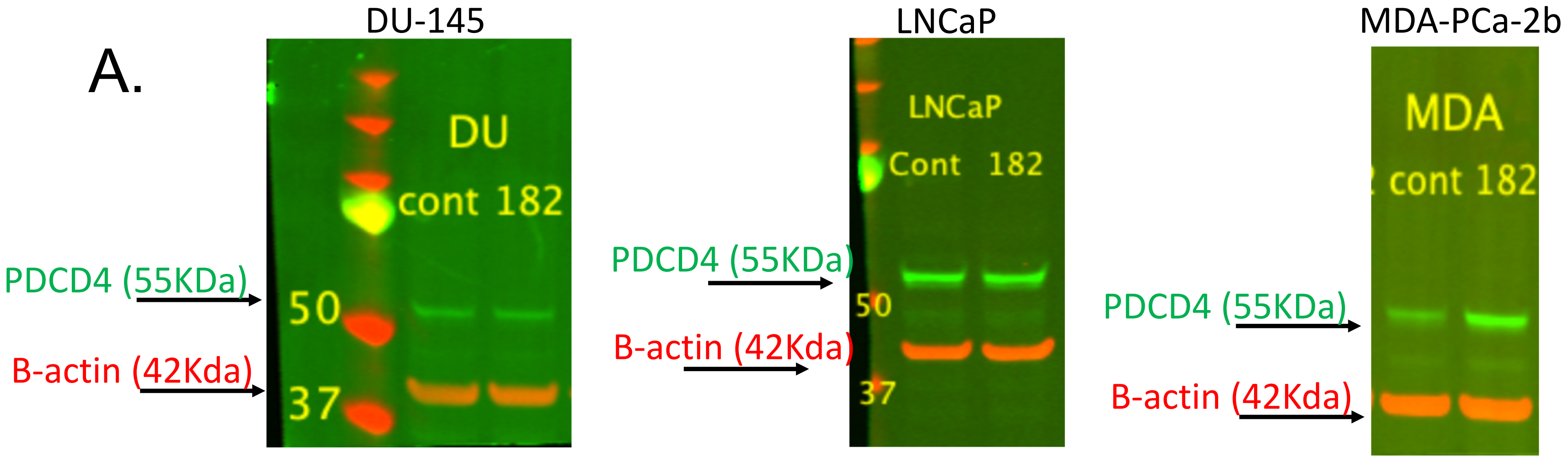

B.

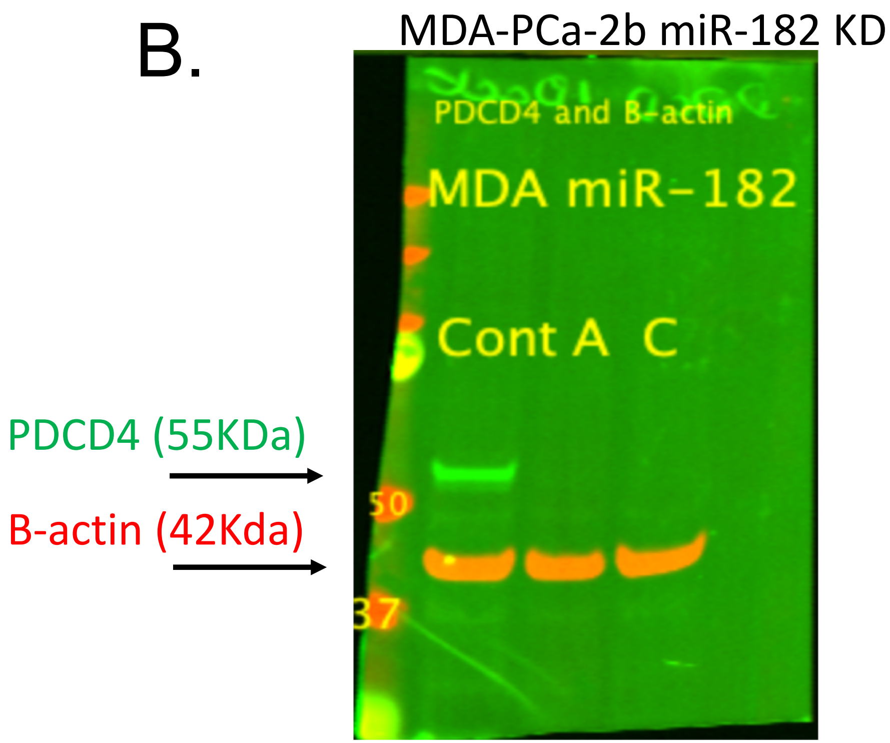

Supplement: Supplementary file 1 — Additional file 1. [file 12885_2021_8723_MOESM1_ESM.pdf]
